# CC8 MRSA Strains Harboring SCC*mec* Type IVc are Predominant in Colombian Hospitals

**DOI:** 10.1371/journal.pone.0038576

**Published:** 2012-06-20

**Authors:** J. Natalia Jiménez, Ana M. Ocampo, Johanna M. Vanegas, Erika A. Rodriguez, José R. Mediavilla, Liang Chen, Carlos E. Muskus, Lázaro A. Vélez, Carlos Rojas, Andrea V. Restrepo, Sigifredo Ospina, Carlos Garcés, Liliana Franco, Pablo Bifani, Barry N. Kreiswirth, Margarita M. Correa

**Affiliations:** 1 Grupo de Microbiología Molecular, Escuela de Microbiología, Universidad de Antioquia, Medellín, Colombia; 2 Public Health Research Institute, University of Medicine and Dentistry of New Jersey, Newark, New Jersey, United States of America; 3 Programa de Estudio y Control de Enfermedades Tropicales-PECET, Universidad de Antioquia, Medellín, Colombia; 4 Grupo Investigador de Problemas en Enfermedades Infecciosas-GRIPE, Universidad de Antioquia, Medellín, Colombia; 5 Grupo de Epidemiología, Universidad de Antioquia, Medellín, Colombia; 6 Hospital Pablo Tobón Uribe, Medellín, Colombia; 7 Hospital Universitario San Vicente Fundación, Medellín, Colombia; 8 Clinica Cardiovascular, Congregación Mariana, Medellín, Colombia; 9 Tuberculosis and Mycobacteria Communicable & Infectious Diseases, Scientific Institute of Public Health, Brussels, Belgium; Rockefeller University, United States of America

## Abstract

**Background:**

Recent reports highlight the incursion of community-associated MRSA within healthcare settings. However, knowledge of this phenomenon remains limited in Latin America. The aim of this study was to evaluate the molecular epidemiology of MRSA in three tertiary-care hospitals in Medellín, Colombia.

**Methods:**

An observational cross-sectional study was conducted from 2008–2010. MRSA infections were classified as either community-associated (CA-MRSA) or healthcare-associated (HA-MRSA), with HA-MRSA further classified as hospital-onset (HAHO-MRSA) or community-onset (HACO-MRSA) according to standard epidemiological definitions established by the U.S. Centers for Disease Control and Prevention (CDC). Genotypic analysis included SCC*mec* typing, *spa* typing, PFGE and MLST.

**Results:**

Out of 538 total MRSA isolates, 68 (12.6%) were defined as CA-MRSA, 243 (45.2%) as HACO-MRSA and 227 (42.2%) as HAHO-MRSA. The majority harbored SCC*mec* type IVc (306, 58.7%), followed by SCC*mec* type I (174, 33.4%). The prevalence of type IVc among CA-, HACO- and HAHO-MRSA isolates was 92.4%, 65.1% and 43.6%, respectively. From 2008 to 2010, the prevalence of type IVc-bearing strains increased significantly, from 50.0% to 68.2% (*p = *0.004). Strains harboring SCC*mec* IVc were mainly associated with *spa* types t1610, t008 and t024 (MLST clonal complex 8), while PFGE confirmed that the t008 and t1610 strains were closely related to the USA300-0114 CA-MRSA clone. Notably, strains belonging to these three *spa* types exhibited high levels of tetracycline resistance (45.9%).

**Conclusion:**

CC8 MRSA strains harboring SCC*mec* type IVc are becoming predominant in Medellín hospitals, displacing previously reported CC5 HA-MRSA clones. Based on shared characteristics including SCC*mec* IVc, absence of the ACME element and tetracycline resistance, the USA300-related isolates in this study are most likely related to USA300-LV, the recently-described ‘Latin American variant’ of USA300.

## Introduction

Since its emergence in 1961, methicillin-resistant *Staphylococcus aureus* (MRSA) has traditionally been considered a nosocomial pathogen. In recent years, however, community-associated MRSA (CA-MRSA) has emerged as an important global public health problem [1]. Healthcare-associated MRSA (HA-MRSA) infections generally occur in individuals with predisposing risk factors such as surgery or presence of indwelling medical devices, whereas CA-MRSA infections typically occur in otherwise healthy individuals who do not exhibit such risk factors [2]. Eleven SCC*mec* types (SCC*mec* I-XI) have been described to date [3], [4]; of these, SCC*mec* types I, II and III are characteristic of traditional HA-MRSA strains, while types IV, V and VI are generally associated with CA-MRSA [1].

In recent years, however, the distinction between HA-MRSA and CA-MRSA has become increasingly blurred, with a growing number of reports indicating that CA-MRSA strains are spreading in hospital settings and replacing traditional HA-MRSA strains [5], [6], [7], [8], [9], [10], [11], [12], [13], [14], [15].

In Colombia, MRSA constitutes an increasingly worrisome clinical problem [5], [12]; however, understanding of its molecular epidemiology remains limited. Several HA-MRSA clones have been described, including the Pediatric clone (ST5-MRSA-IV, currently classified as SCC*mec* type VI [16]), the Brazilian clone (ST239-MRSA-III) and the Chilean clone (ST5-MRSA-I), mainly in large tertiary hospitals in Bogotá, the capital city [17], [18]. In addition, the CA-MRSA clone USA300 (ST8-MRSA-IV) has recently been associated with nosocomial infections in Latin America [5], [12], [19]. Most of the latter isolates belong to a distinct Latin American variant of USA300 recently dubbed “USA300-LV”, which is characterized by carriage of SCC*mec* IVc, absence of the arginine catabolic mobile element (ACME) and high prevalence of tetracycline resistance [12], [20]. Considering the dynamic nature of MRSA epidemiology, it is imperative to evaluate the situation in other regions of Colombia where MRSA constitutes a severe problem. Accordingly, the aim of this study was to evaluate the molecular epidemiology of MRSA strains isolated from patients between 2008–2010 in three tertiary-care hospitals in Medellín, the second largest city in Colombia.

## Methods

### Study Population

An observational cross-sectional study was conducted at three tertiary-care hospitals from February 2008 to June 2010. Hospital A is a large 648-bed university hospital; Hospital B is a 380-bed medium-size tertiary care center; and Hospital C is a 140-bed cardiology hospital. All institutions are located in Medellín, the second largest city in Colombia. Sample sizes were calculated based on the prevalence of MRSA at each institution during 2007 (40.0% for Hospital A, 25.0% for Hospital B and 10.0% for Hospital C). Patients infected with MRSA were recruited prospectively and only the first isolate from each individual was evaluated. A final sample of 538 MRSA isolates was included. The research protocol and informed consent (signed by participants, parents, or guardians) were approved by the Bioethics Committee for Human Research of the University Research Center at Universidad de Antioquia (CBEIH-SIU) (approval No 0841150), as well as by the Ethics Committee in Research of Hospital Universitario San Vicente Fundación, the Ethics Committee in Research of Hospital Pablo Tobón Uribe and the Ethics Committee in Research of Clínica Cardiovascular Congregación Mariana (institutions where participants were recruited).

### Clinical and Epidemiological Data

Clinical and epidemiological information was obtained from medical records for each patient. Infections were classified as either CA- or HA-MRSA, according to standard epidemiological definitions established by the U.S. Centers for Disease Control and Prevention (CDC) [21]. Healthcare-associated infections were further classified as either community-onset (HACO) or hospital-onset (HAHO) [21]. Infections were classified as HACO if (i) positive MRSA culture was obtained within the first 48 hours of hospital admission and (ii) at least one of the following healthcare-associated risk factors was present: presence of an invasive device at time of admission, history of MRSA infection, surgery, hospitalization, dialysis, or intensive care unit (ICU) admission during the 12 months preceding the culture date. Infections were classified as HAHO if (i) positive MRSA culture was obtained 48 hours after hospital admission and (ii) at least one of the above-mentioned risk factors was present. Lastly, infections were defined as community-associated (CA) if positive MRSA culture was obtained during the first 48 hours of hospital admission without healthcare-associated risk factors.

### Laboratory Methods

#### Susceptibility testing

Antibiotic susceptibilities of *S. aureus* isolates were assessed in accordance with Clinical Laboratory Standards Institute (CLSI) guidelines [22]. Antibiotics tested included clindamycin, erythromycin, gentamicin, linezolid, moxifloxacin, oxacillin, rifampin, tetracycline, tigecycline, trimethoprim-sulfamethoxazole and vancomycin. *S. aureus* ATCC 29213 was used as a quality control strain.

#### PCR confirmation of S. aureus and methicillin resistance

Presence of the species-specific *nuc* and *femA* genes, as well as the *mecA* gene (determinant of methicillin resistance), were verified by polymerase chain reaction (PCR) as previously described [23], [24].

#### SCCmec typing

SCC*mec* types and subtypes were determined using two previously described sets of multiplex PCR reactions [25], [26]. MRSA strains used as positive controls for SCC*mec* types and SCC*mec* IV subtypes were kindly provided by Dr. Teruyo Ito (Juntendo University, Japan) and included: NCTC10442 (SCC*mec* I), N315 (SCC*mec* II), 85/2082 (SCC*mec* III), JCSC4744 (SCC*mec* IVa), JCSC2172 (SCC*mec* IVb), 81/108 (SCC*mec* IVc), JCSC4469 (SCC*mec* IVd), JCSC4796 (SCC*mec* IVg) and WIS (SCC*mec* V).

**Table 1:** Classification of MRSA infections according to CDC epidemiological criteria.

Hospital^a^	CA-MRSA^b^ No. (%)	HA-MRSA^c^		Total no. (%)
		HACO-MRSA^d^ No. (%)	HAHO-MRSA^e^ No. (%)	
A	46 (13.3)	133 (38.6)	166 (48.1)	345 (100.0)
B	21 (13.5)	93 (59.6)	42 (26.9)	156 (100.0)
C	1 (2.7)	17 (45.9)	19 (51.4)	37 (100.0)
**Total**	68 (12.6)	243 (45.2)	227 (42.2)	538 (100.0)

a A: Largest hospital, B: Medium-sized hospital, C: Cardiologic hospital.

b CA-MRSA: community-associated methicillin-resistant *Staphylococcus aureus.*

c HA-MRSA: healthcare-associated methicillin-resistant *Staphylococcus aureus.*

d HACO-MRSA: healthcare-associated, community-onset MRSA.

e HAHO-MRSA: healthcare-associated, hospital-onset MRSA.

#### Spa typing and multilocus sequence typing (MLST)

The polymorphic X region of the protein A gene (*spa*) was amplified and sequenced as previously described [27]. Corresponding *spa* types were assigned using eGenomics software, as described previously [27], [28]. Ridom *spa*-types were subsequently assigned using the *spa* typing website (http://www.spaserver.ridom.de/) developed by Ridom GmbH and curated by SeqNet.org (http://www.SeqNet.org/) [29]. MLST was performed on a representative subset of 54 isolates (∼10% of all samples) using the methodology described by Enright *et al*. [30]. Allele numbers and sequence types (ST)s were assigned using the database maintained at http://saureus.mlst.net/, while clonal complexes (CC)s were inferred using eBURST analysis [31]. Clonal complexes for all remaining strains were inferred by *spa* repeat pattern analysis [28], or by referring to the Ridom SpaServer website.

**Figure 1:**
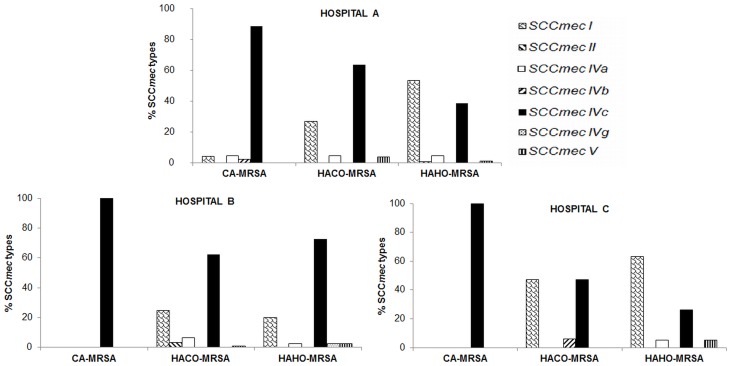
SCC*mec* types of MRSA-isolates from each hospital, classified according to CDC epidemiological criteria. Abbreviations: CA-MRSA: community-associated methicillin-resistant *Staphylococcus aureus*; HACO-MRSA: healthcare-associated, community-onset methicillin-resistant *Staphylococcus aureus;* HAHO-MRSA: healthcare-associated, hospital-onset methicillin-resistant *Staphylococcus aureus*.

#### Pulsed-field gel electrophoresis (PFGE)

PFGE following *Sma*I digestion was performed according to a protocol described elsewhere [32]. DNA fragment patterns were normalized using *S. aureus* strain NCTC8325. Band assignments were manually adjusted after automatic band detection and only bands ranging from 36 kb to 600 kb were utilized for analysis. Cluster analysis was performed using the Dice coefficient in BioNumerics software version 6.0 (Applied Maths, Sint-Martens-Latem, Belgium). Dendrograms were generated by the unweighted pair group method using average linkages (UPGMA), with 1% tolerance and 0.5% optimization settings. Similarity cutoffs of 80% and 95% were used to define types and subtypes, respectively [32]. Representatives of the most common MRSA clones described in Colombia (Chilean, Pediatric and USA300) were used as reference strains.

**Table 2:** Molecular characteristics of MRSA isolates.

MLST- clonal complex	*spa* type^a^	SCC*mec* type^a^	n (%)^b^
CC 1	t922	IVc	2 (0.4)
CC5	**t149^d^, t7279^d^, t1311^d^**, t045, t143, t458^d^,t653, t5756, t7275, t7280^d^	**I**	**166 (31.9)**
	t002	II	3 (0.6)
	t045^d^, t002	IV^c^	5 (1.0)
	t002	IVa	4 (0.8)
	t002^d^, t045	IVc	5 (1.0)
CC 8	t1610, t304	I	5 (1.0)
	t008	II	1 (0.2)
	t1610	IV^c^	1 (0.2)
	t1635^e^, t008^f^	IVa	17 (3.3)
	**t1610^f^, t008^f^, t024^f^**, t051, t068, t203, t301^f^, t304, t574^f^, t622, t656, t770, t2849, t2953, t3060, t4146, t5751, t6442, t6869, t7277, t7278, t8746	**IVc**	**286 (54.9)**
CC9	t209	V	1 (0.2)
CC15	t1885	IVc	1 (0.2)
	t228	V	1 (0.2)
CC30	t021^g^, t012	IVc	4 (0.8)
	t021	IVg	1 (0.2)
CC45	t050	I	2 (0.4)
	t5211	IVc	1 (0.2)
CC59	t216	IVc	1 (0.2)
	t216^h^	V	3 (0.6)
CC72	t148^i^, t324	V	5 (1.0)
CC88	t2649	IVa	1 (0.2)
	t5916	IVb	1 (0.2)
CC97	t267	IVc	1 (0.2)
CC101	t056	IVc	1 (0.2)
CC152	t355	I	1 (0.2)
CC188	t189^j^	IVc	1 (0.2)

a The most prevalent *sp*a type and SCC*mec* type are shown in boldface.

b Number and percentage of isolates with a specific clonal complex (CC) and SCC*mec* type combination.

c SCC*mec* IV subtype could not be determined.

d,e,f,g,h,i,j MLST was performed on representative strains bearing these *spa* types, with the following results: ^d^ST5 (*n* = 15), ^e^ST923 (*n* = 2), ^f^ST8 (*n* = 33), ^g^ST30 (*n* = 1) ^h^ST59 (*n* = 1) ^i^ST72 (*n* = 1) and ^j^ST188 (*n* = 1) (Table S1).

**Figure 2:**
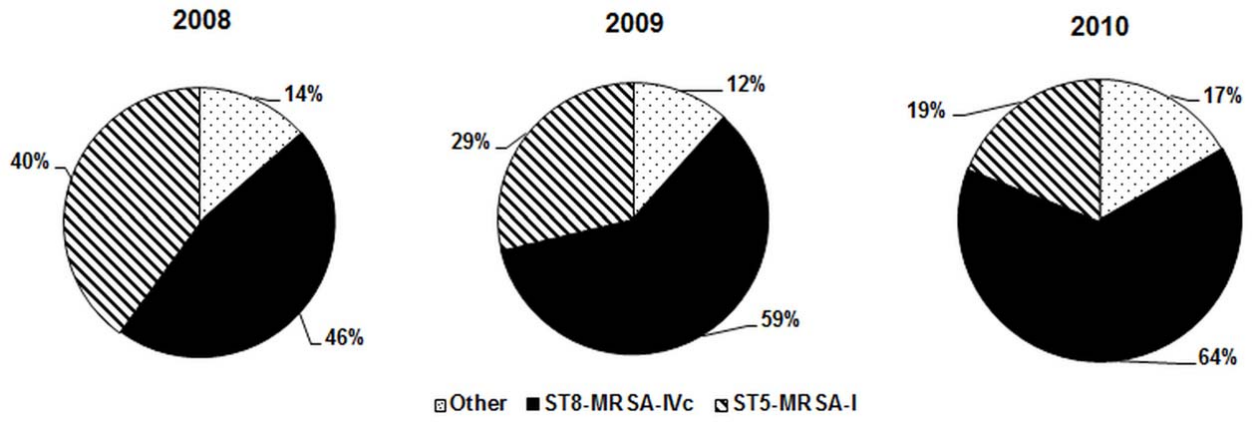
Changing patterns in the prevalence of ST8-MRSA-IVc and ST5-MRSA-I isolates from 2008 to 2010. Other: MRSA isolates with different sequence type (ST) and/or SCC*mec* type.

#### Detection of staphylococcal virulence factors

Genes encoding staphylococcal enterotoxins (*sea, seb, sec, sed, see*), toxic shock syndrome toxin 1 (*tst*) and exfoliative toxins A and B (*eta, etb*) were detected by multiplex PCR as previously described [24]. The *lukS/F-PV* genes encoding Panton-Valentine leukocidin (PVL), as well as the *arcA* gene from the USA300-associated arginine catabolic mobile element (ACME), were also assayed by PCR [33], [34].

#### Statistical analyses

Categorical variables were compared using Chi-square or Fisher’s exact tests, with *p* values ≤0.05 considered statistically significant. Statistical analyses were carried out using the software package SPSS® v15.0 (SPSS Inc., Chicago, USA).

**Figure 3:**
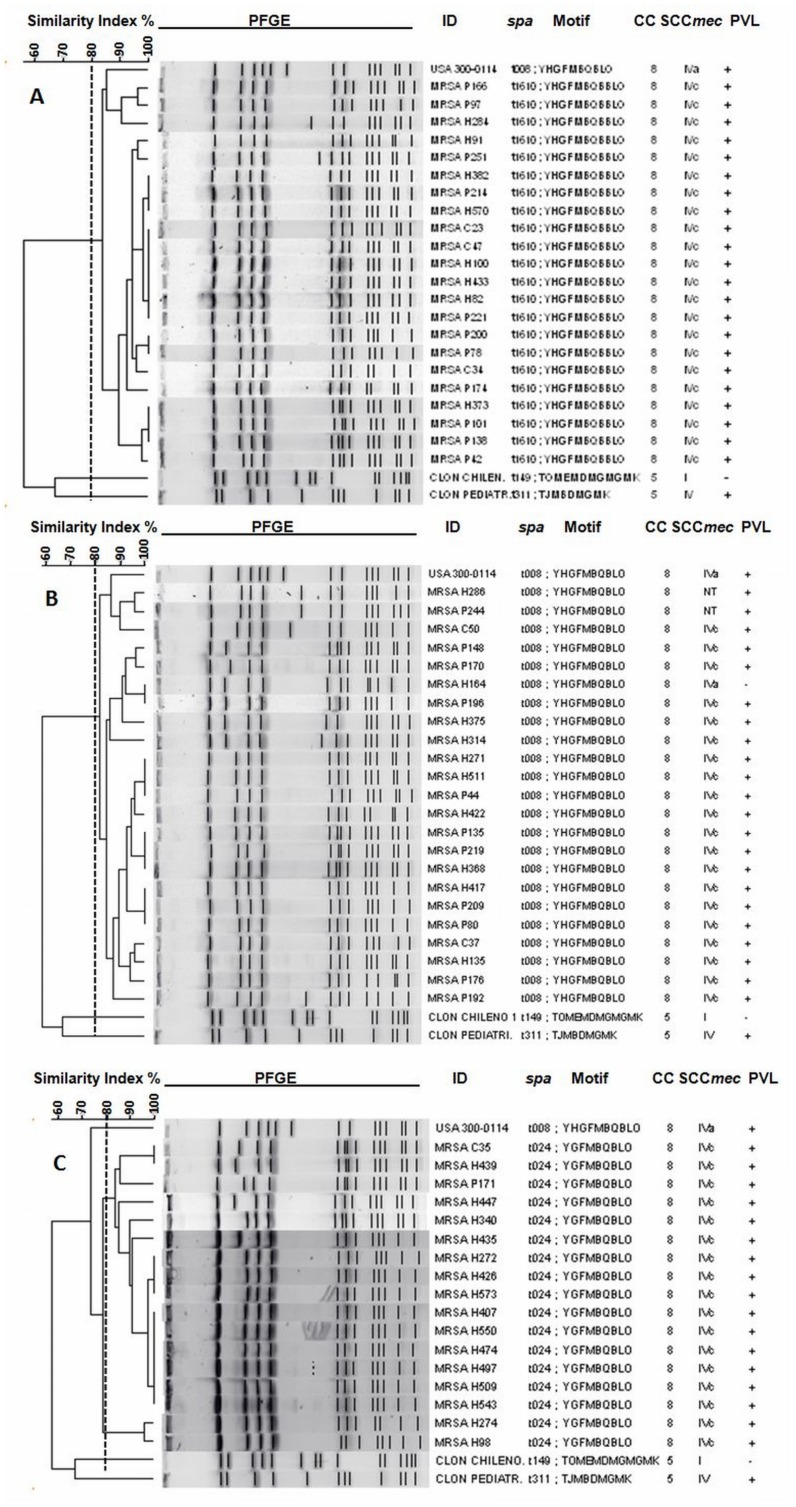
Genetic relatedness between CC8 MRSA isolates, stratified by *spa* type. UPGMA dendrogram showing genetic relatedness between representative CC8 MRSA isolates in this study, as determined by PFGE with *Sma*I and stratified according to *spa* type: (A) *spa* type t1610 isolates, (B) *spa* type t008 isolates and (C) *spa* type t024 isolates. The broken line corresponds to the cutoff level (80%) used to define related PFGE clones.

**Figure 4:**
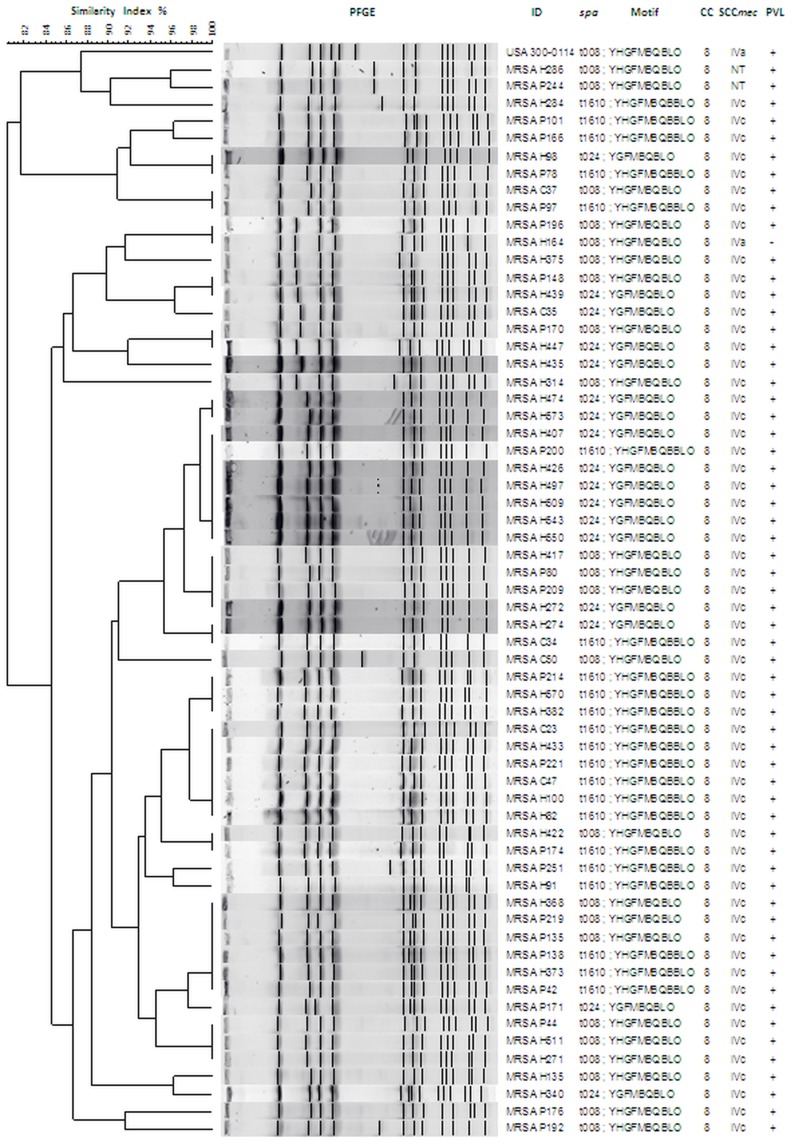
Genetic relatedness between CC8 MRSA isolates, without stratification. UPGMA dendrogram showing genetic relatedness between representative CC8 MRSA isolates in this study as determined by PFGE with *Sma*I, without stratification by *spa* type. Note that t008, t024 and t1610 isolates do not cluster separately by PFGE.

## Results

### Clinical and Epidemiological Data

A total of 538 MRSA isolates were obtained from patients admitted to all three hospitals, including 345 patients from Hospital A, 156 patients from Hospital B and 37 patients from Hospital C. Among the patients included in the study, 355 (66.0%) were males, while the median age among all patients was 39 years (range 0 to 92 years). Using CDC criteria, 243 (45.2%) isolates were classified as HACO-MRSA, 227 (42.2%) as HAHO-MRSA and 68 (12.6%) as CA-MRSA. Variations in the prevalence of the above classifications were found in each institution evaluated (Table 1). Hospitals A and C had the highest prevalence of HAHO-MRSA (48.1% and 51.4% respectively), while Hospital B had the highest prevalence of HACO-MRSA (59.6%).

**Figure 5:**
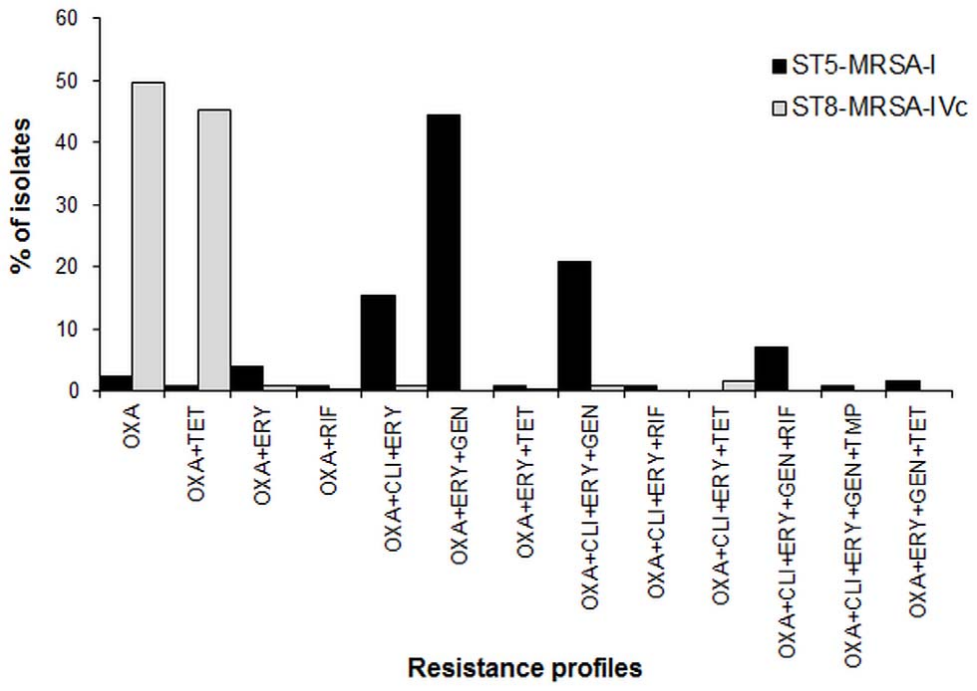
Antibiotic resistance profiles of isolates from each hospital according to MRSA genotype. Abbreviations: CLI, clindamycin; ERY, erythromycin; GEN, gentamicin; OXA, oxacillin; RIF, rifampin; TET, tetracycline; TMP, trimethroprim-sulfamethoxazole.

Most of the MRSA isolates were obtained from patients admitted to the surgery department (22.6%), followed by internal medicine (19.9%), orthopedics (19.7%), pediatrics (14.1%), ICU (9.3%) and other medical services (8.9%). Isolates were most commonly obtained from skin and soft tissue infections (40.8%), followed by surgical site infections (16.2%), osteomyelitis (12.4%), pneumonia (10.4%), central venous catheter-related bacteremia (7.9%), primary bacteremia (5.8%) and other infections (6.5%) including arthritis, urinary tract infections, conjunctivitis, meningitis, otitis, tracheitis, orbital cellulitis and infected pulmonary sequestration.

### Characterization of MRSA Strains

Of the 538 MRSA isolates analyzed by multiplex PCR [25], the SCC*mec* types of 521 (96.8%) could be determined. Seven different SCC*mec* types and subtypes were detected, including types I, II, IVa, IVb, IVc, IVg and V. Among the 17 remaining isolates, 11 were non-typeable for SCC*mec* type and 6 were non-typeable for SCC*mec* IV subtype. The majority of the typeable MRSA isolates harbored SCC*mec* type IVc (306, 58.7%) followed by SCC*mec* type I (174, 33.4%).The remaining SCC*mec* types were present at very low frequency, including types II (4, 0.8%), IVa (23, 4.4%), IVb (2, 0.4%), IVg (1, 0.2%) and V (10, 1.9%), while SCC*mec* type III was not detected at all. Isolates classified as CA-MRSA and HACO-MRSA most commonly harbored SCC*mec* type IVc (92.4% and 65.1%, respectively), while HAHO-MRSA strains mainly harbored SCC*mec* type I (48.2%) in addition to SCC*mec* type IVc (43.6%). Notably, SCC*mec* type IVc was the most frequent subtype found in HAHO-MRSA (72.5%) from Hospital B (Figure 1), suggesting that strains harboring SCC*mec* IVc have become a major source of HA- as well as CA-MRSA infections.

All of the MRSA isolates were represented by 53 different *spa* types (Table 2), with the most common types being t1610 (eGenomics 814, YHGFMBQBBLO, 25.6%), t149 (eGenomics 442, TO2MEMDMGMGMK, 20.9%), t008 (eGenomics 1, YHGFMBQBLO, 12.6%) and t024 (eGenomics 363, YGFMBQBLO, 10.7%). Among the 53 *spa* types, a total of 12 MLST clonal complexes were found, with CC8 and CC5 accounting for 60.8% and 34.1% of the isolates, respectively. Isolates belonging to *spa* types t1610, t008 and t024 corresponded to CC8 and mainly harbored SCC*mec* type IVc (99.2%), whereas the t149 isolates corresponded to CC5 and harbored SCC*mec* type I exclusively (Table 2, Table S1). Over the three year course of the study, ST8 isolates harboring SCC*mec* type IVc (primarily *spa* types t1610, t008 and t024) increased significantly in frequency, from 46.5% in 2008 to 64.3% in 2010 (*p*<0.05). By contrast, ST5 isolates harboring SCC*mec* type I decreased in frequency, from 39.9% in 2008 to 19.0% in 2010 (*p*<0.05) (Figure 2).

Twenty isolates from each of the most predominant CC8 *spa* types were further analyzed by PFGE (Figure 3). Isolates belonging to *spa* type t1610 and t008 were closely related to the USA300-0114 CA-MRSA clone (ST8-MRSA-IVa), with similarity coefficients ranging from 80–85%, whereas the t024 isolates did not appear to be related to this strain (similarity coefficient 73%). However, when PFGE was performed simultaneously on all three *spa* types, they all appeared to be closely related to USA300-0114 (>80% similarity coefficient) (Figure 4). Moreover, they did not cluster separately by *spa* type when analyzed together. As expected, the *spa* type t149 (CC5) isolates were closely related (similarity coefficients ranging from 85–97%) to the Cordobes/Chilean clone (ST5-MRSA-I), whereas isolates related to the previously reported Pediatric clone (ST5-MRSA-IV, now classified as SCC*mec* type VI) were not found (data not shown).

The isolates displayed distinct virulence gene patterns according to clonal complex and SCC*mec* type, with CC8 isolates harboring SCC*mec* types IVa, IVc and V more likely to possess genes coding for virulence factors. The genes for PVL (*lukS/F-PV*) were present in isolates harboring most SCC*mec* types (except types II and IVg) and were most frequently observed in isolates harboring SCC*mec* type IVc (90.6%). Exfoliative toxin gene A (*eta*) was only detected in a single CC8 strain harboring SCC*mec* type V, while exfoliative toxin gene B (*etb*) was not detected at all. Various staphylococcal enterotoxin genes were also associated with different SCC*mec* types, with *seb* and *sed* being the most common. The ACME-*arcA* gene, frequently associated with USA300-0114 strains, was not detected in any of the isolates tested.

Antibiotic resistance to clindamycin, erythromycin, gentamicin, moxifloxacin and tetracycline differed significantly among MRSA isolates carrying SCC*mec* type I vs. SCC*mec* type IVc (*p<*0.0001). ST5-MRSA-I isolates were resistant to clindamycin (90.1%), erythromycin (94.8%), gentamicin (88.7%), moxifloxacin (28.7%), rifampin (12.1%), tetracycline (4.6%) and trimethoprim-sulfamethoxazole (2.0%), whereas ST8-MRSA-IVc displayed much lower resistance to clindamycin (3.6%), erythromycin (4.9%), gentamicin (1.0%), moxifloxacin (0.5%), rifampin (0.7%) and trimethoprim-sulfamethoxazole (0.8%), but significantly higher resistance to tetracycline (45.9%). Overall, ST5-MRSA-I exhibited four principal antimicrobial resistance patterns, whereas ST8-MRSA-IVc exhibited two patterns (Figure 5). Without exception, all MRSA isolates were susceptible to vancomycin, linezolid and tigecycline.

## Discussion

Molecular epidemiology studies have highlighted the continuing global evolution and spread of different MRSA clones [35]. The factors contributing to dissemination of MRSA clones are only partially understood, but are thought to include migration of human populations, ineffective methods to control MRSA transmission and inadequate treatment strategies, including the inappropriate selection and use of antibiotics [36]. Since 2003, many reports have documented the spread of SCC*mec* type IV-harboring CA-MRSA strains in hospital settings, primarily in Europe and the United States (U.S.), but also in South America [5], [6], [7], [8], [9], [10], [12], [13], [14], [15]. Partly as a result of this incursion, the epidemiologic distinction between CA-MRSA and HA-MRSA has become increasingly blurred. Understanding of this phenomenon remains limited in Latin America, mostly due to a relative lack of studies documenting the current epidemiology of MRSA in institutions within these countries. Accordingly, this study focuses on the epidemiology of MRSA in three important tertiary-care hospitals in Medellín, Colombia.

Our findings confirm a high prevalence of SCC*mec* type IVc-bearing isolates (traditionally associated with CA-MRSA) in local hospitals, where they are currently circulating as nosocomial pathogens along with HA-MRSA strains harboring SCC*mec* type I.

In this study, SCC*mec* type IVc was predominant in isolates classified as CA-MRSA (92.4%) and HACO-MRSA (65.1%), while among HAHO-MRSA the frequency of SCC*mec* type IVc (43.6%) was nearly as high as that of SCC*mec* type I (48.2%). Moreover, differences in the prevalence of SCC*mec* type IVc were found among the three hospitals in our study. For example, in Hospital B, SCC*mec* IVc was found more frequently among HAHO-MRSA isolates (72.5%), whereas in Hospital C, HA-MRSA isolates (both HACO and HAHO) mainly harbored SCC*mec* type I (47.1% and 54.7%, respectively). In our study, the emergence of SCC*mec* type IVc in hospital settings was primarily observed in CC8 strains exhibiting three *spa* types (t1610, t008, t024). Data collected over the course of the study highlight the expansion of these strains, which increased in prevalence from 2008 (46.5%) to 2010 (64.3%).

SCC*mec* type IVc has been found in other widely disseminated CA-MRSA clones, such as the Oceania-South West Pacific clone (ST30-MRSA-IVc), which was initially described in New Zealand [37] and subsequently isolated from nearly every continent [2]. In South America, it has been reported in CC30 strains from Brazil, Uruguay and, more recently, Argentina [6], [13], [38], [39], [40], [41], while several reports have described an SCC*mec* type IVc-bearing variant of USA300 in Colombia, Ecuador, Venezuela, Peru, Argentina and Trinidad and Tobago [5], [20]. This Latin American variant, recently dubbed “USA300-LV” [20], is distinguished from prototypical USA300-0114 strains in that it harbors SCC*mec* IVc rather than IVa, lacks the ACME element and is frequently resistant to tetracycline. Although USA300-LV control strains [12] were not available for comparative analysis, the SCC*mec* IVc-harboring USA300-related strains in our study also possessed these characteristics and are most likely representatives of the USA300-LV lineage.

Currently, t008 (CC8) is the fourth most common *spa* type in the Ridom database (5.93%) and is represented in most European countries, Argentina, Canada and especially the U.S., where it accounts for the majority of USA300 CA-MRSA isolates [1]. It is also the primary *spa* type associated with USA300-LV, which has been reported in several countries outside of Latin America, including Spain, Italy, Belgium, the Netherlands, the United Kingdom and Australia [20]. *Spa* type t024 (CC8), while less common than t008 (0.78% in Ridom), is also widespread globally, particularly in Denmark [1]. By contrast, in our study the most common *spa* type (25.7%) among all 538 MRSA isolates was t1610 (CC8), which has been isolated in Spain, Germany, Sweden, Norway, Canada and the U.S., but has a low overall frequency in the Ridom database (0.01%). Lastly, t149 (CC5), associated primarily with the SCC*mec* type I isolates in our study, is one of the most common *spa* types in South America, including Argentina, Brazil, Chile and Paraguay and is related to the previously described Cordobes/Chilean clone [42], [43].

Overall, the single most prevalent lineage in this study was ST8-MRSA-IVc (55.0%), followed by ST5-MRSA-I (31.7%). By contrast, an earlier report from Colombia [18] found that MRSA strains isolated from 1996–1998 at five hospitals in Bogotá displayed 80% homology to the Pediatric clone (ST5-MRSA-IV, currently classified as VI), which is characterized by a multi-resistant phenotype. However, this lineage was completely absent from the isolates analyzed in this study. In a subsequent study conducted by Cruz *et al*. [17], the Cordobes/Chilean clone (ST5-MRSA-I) was shown to be the dominant nosocomial strain from 2001–2003. More recently, an ST8-MRSA-IV clone related to USA300 (*i.e*., USA300-LV) was described as causing CA-MRSA infections in Colombia [44], with reports of nosocomial infections surfacing in 2009 and 2010 [5], [12]. In this study, the prevalence of the ST8-MRSA-IVc clone appeared to increase over time, such that presently it is the predominant clone causing both CA-MRSA as well as HA-MRSA infections.

The most prevalent lineages observed in our study were notably different from those found in other South American regions, where ST5-MRSA-II, ST5-MRSA-IV, ST30-MRSA-IV and ST239-MRSA-III appear to predominate [19]. In addition, the Cordobes/Chilean ST5-MRSA-I clone, highly prevalent in South American HA-MRSA infections, is being displaced by the ST8-MRSA-IVc clone in Medellín. These results highlight the geographic diversity among MRSA strains and the predominantly clonal evolution of healthcare-associated MRSA in Colombia, with successive clonal replacement observed over time. Moreover, they emphasize the importance of local surveillance and dissemination of findings, in order to ensure that medical personnel are aware of changing patterns of MRSA epidemiology within their institutions, thereby enabling them to choose efficacious empirical treatments. In addition, these results, along with similar reports from hospitals in various different countries [5], [32], [33], [45], [46], suggest that the use of SCC*mec* type IV as a genetic marker for CA-MRSA is of increasingly limited significance.

The differences observed in *spa* typing and virulence gene content, in conjunction with the results of PFGE, suggest the presence of considerable genetic diversity among the ST8-MRSA-IVc strains that have become established in Medellín. In this study, we performed *spa* typing prior to PFGE, then compared different *spa*-based PFGE clusters to USA300-0114, as well as to other clones previously reported in Colombia. As shown in Figure 3, whereas the t1610 and t008 isolates were closely related to USA300-0114 (>80% similarity), the t024 isolates did not appear to be related (similarity coefficient 73%). However, when analyzed together (Figure 4), all three *spa* types were closely related to USA300-0114 by PFGE (>80% similarity) and did not cluster separately. These results are similar to those of a previous study from Denmark [45], in which t008 and t024 isolates were indistinguishable by PFGE. In this study, the t024 strains were genetically distinct from USA300-0114 by PFGE, but possessed the same characteristics as t1610 and t008, including SCC*mec* IVc, absence of ACME and tetracycline resistance. It is therefore likely that all three ST8-MRSA-IVc *spa* types belong to the USA300-LV lineage. Nevertheless, in accordance with Larsen *et al*’s recommendations [45], *spa* typing allowed for differentiation of clones which could not be discriminated using PFGE alone. These findings are relevant since studies performed in Colombia and South America typically report the dissemination of USA300 based solely on PFGE [5], [12], whereas our results also suggest that additional methods such as *spa* typing should be performed to differentiate genetically and epidemiologically distinct strains.

Taken together, the data from this study suggest that CC8 MRSA strains harboring SCC*mec* IVc are becoming predominant in Medellín hospitals. In addition to the previously described *spa* type t008 USA300 strains [5], [12], two novel ST8-MRSA-IVc *spa* types (t024 and t1610) have been identified for the first time in Latin America. Given the transmissibility and potential virulence of CA-MRSA strains, continued increase in the prevalence, severity and complexity of infections caused by these emerging clones may lead to greater morbidity, mortality and hospitalization costs [11], [47]. Rigorous surveillance is therefore necessary in Colombia and Latin America, in order to heighten awareness of contemporary MRSA clones circulating in specific countries and institutions and mitigate their impact on the design of effective strategies for controlling MRSA transmission. Unfortunately, individual hospitals within Colombia have typically implemented their own infection control policies. Recently, however, the National Institute of Health has started implementing a national surveillance program for healthcare-related infections, including antimicrobial consumption and resistance patterns, within hospitals.

## Supporting Information

Table S1 **Results of MLST in representative strains of major** ***spa*** **types.**
(XLS)
